# Serial dependence does not build up to central tendency bias in orientation judgments

**DOI:** 10.1167/jov.26.10.4

**Published:** 2026-10-02

**Authors:** Saija M. Niemi, Toni P. Saarela, Maria Olkkonen

**Affiliations:** 1 Department of Psychology, Faculty of Medicine, University of Helsinki, Helsinki, Finland

**Keywords:** central tendency bias, serial dependence, orientation perception, sequential effects, circular stimulus space

## Abstract

Perception is shaped by sequential biases operating on multiple timescales, including short-term serial dependence, medium-term central tendency bias (CTB), and long-term effects like the cardinal bias in orientation judgments. Although serial dependence has been widely observed in orientation judgments, CTB has not been reliably demonstrated despite its prevalence in other stimulus types. In this study, we investigated how these biases co-occur by comparing orientation judgments in two conditions: one with stimuli sampled from a full circular uniform distribution and another from a restricted section of orientations. Participants exhibited a cardinal bias away from cardinal orientations and an attractive serial dependence in both conditions. Contrary to expectations, no CTB was observed in the section condition independent of cardinal bias. Instead, participants showed a repulsive bias away from the center of the stimulus distribution, independent of cardinal biases. This repulsive bias persisted after controlling for serial dependence, suggesting that it may reflect a medium-term effect rather than a statistical artifact. The results challenge models that posit a shared mechanism for CTB and serial dependence and show that attractive serial dependence is not sufficient for a CTB to emerge. These findings highlight the need to account for stimulus space geometry and both attractive and repulsive sequential biases.

## Introduction

The information extracted by the human visual system about the external environment is unavoidably noisy. Sensory signals are corrupted by, for example, optical factors, movement of objects and the observer, and inherent neuronal noise. Yet, we experience the world in a mostly stable and valid manner that allows us to make accurate-enough judgments to act in it. One method for countering the noise in sensory signals is to combine and complete these signals with previously acquired information (Burr & Cicchini, 2014; Corbett, Fischer, & Whitney, 2011; Manassi & Whitney, 2024). In fact, stimulus history affects perceptual estimates on different time scales: from biases that arise from stimuli seen a few seconds ago (Corbett et al., 2011) to learning the statistical properties of the world over the course of a lifetime (Girshick, Landy, & Simoncelli, 2011; Olkkonen, Hansen, & Gegenfurtner, 2008). How these effects relate to each other remains relatively unknown because the research traditions have been largely separate. Here we study the concurrent existence of these effects in orientation perception.

Serial dependence is a short-term sequential effect, where perceptual judgments about the current stimulus are biased toward recently seen, now irrelevant stimuli (Corbett et al., 2011; Fernberger, 1920; Fischer & Whitney, 2014; Holland & Lockhead, 1968). Often, serial dependence exhibits feature tuning: the attraction toward the preceding stimulus follows a curvilinear pattern such that, when the preceding and current stimuli are similar, the bias is maximal, but when the difference grows larger, the attractive bias diminishes or even turns repulsive (Bliss, Sun, & D’Esposito, 2017; Fischer & Whitney, 2014; Manassi, Murai, & Whitney, 2023; Pascucci et al., 2023). Serial dependence has been observed for many different stimulus types in vision, for example, orientation (Fischer & Whitney, 2014), spatial location (Bliss et al., 2017), color (Barbosa & Compte, 2020), motion direction (Czoschke, Fischer, Beitner, Kaiser, & Bledowski, 2019), heading direction (Sun, Zhang, Alais, & Li, 2020), numerosity (Fornaciai & Park, 2018), line length (Saarela, Niemi, & Olkkonen, 2023), visual variance (Suárez-Pinilla, Seth, & Roseboom, 2018), facial identity (Liberman, Fischer, & Whitney, 2014), and facial attractiveness (Xia, Leib, & Whitney, 2016). Additionally, outside vision, it has been found in estimations of odor intensity and familiarity (Van der Burg, Toet, Brouwer, & van Erp, 2022), noise loudness (Holland & Lockhead, 1968), and time interval length (Glasauer & Shi, 2022).

Medium timescale effects manifest over the course of an experiment. Central tendency bias (CTB) describes an effect where perceptual judgments are biased toward the mean of the stimulus distribution used in an experiment (Hollingworth, 1910; Huttenlocher, Hedges, & Vevea, 2000). CTB usually manifests as a linear effect, such that stimuli with lower objective values in the stimulus distribution tend to be overestimated, while those with higher objective values tend to be underestimated (Huttenlocher et al., 2000). In Bayesian observer models, this is conceptualized as the visual system learning the stimulus distribution used in the experiment and using it as the prior, leading to a bias toward the mean of the distribution (Ashourian & Loewenstein, 2011; Jazayeri & Shadlen, 2010). Equal to serial dependence, CTB has been found for numerous different stimulus types, including size (Hollingworth, 1910), line length (Huttenlocher et al., 2000), numerosity (Xiang, Graeber, Enke, & Gershman, 2021), color (Olkkonen, McCarthy, & Allred, 2014), lightness (Olkkonen, Saarela, & Allred, 2016), time interval (Jazayeri & Shadlen, 2010), and noise pitch (Raviv, Ahissar, & Loewenstein, 2012). In orientation perception, because the mean or the center is not defined for circular uniform distributions, unsurprisingly, CTB in orientation judgments was not found when orientations were sampled from a circular distribution (Xiang et al., 2021; Yildirim, Semizer, & Boduroglu, 2024).

In addition to history effects arising in the course of an experiment, perceptual reports also exhibit biases that arise from learning the statistical properties of the world or that are possibly hard-coded in the visual system. An example of this is the effect of high-level visual memory on color perception: memory modulates the perceived chromaticity of familiar objects (Hansen, Olkkonen, Walter, & Gegenfurtner, 2006; Olkkonen et al., 2008). In orientation perception, cardinal bias is a perceptual bias caused by cardinal orientations: horizontal and vertical orientations. Cardinal bias can be attractive, toward cardinal orientations (Girshick et al., 2011), or more commonly repulsive, away from cardinal orientations (Bae, 2022; de Gardelle, Kouider, & Sackur, 2010; Jastrow, 1892; Lennie, 1971; Tomassini, Morgan, & Solomon, 2010). Whether this bias is repulsive or attractive is likely to depend on the task, noise, and stimulus statistics (Bae, 2021; Wei & Stocker, 2015). The repulsive bias is related to a phenomenon called the oblique effect, where judgments about orientations near cardinal orientations are more precise due to more cells in the visual cortex encoding cardinal orientations and those cells having narrower tuning curves (Appelle, 1972; Li, Peterson, & Freeman, 2003). Cardinal bias can also be classified into two separate effects: a low-level bias caused by this neuronal anisotropy and a more cognitive bias (Balikou et al., 2015).

A few recent studies have investigated the relationship between serial dependence and CTB. We previously showed that the preceding stimuli one to six trials back and the cumulative mean of all stimuli have independent effects on human participants’ responses in a two-interval forced-choice task using hue and line length (Saarela et al., 2023). That is, neither serial dependence nor CTB alone can explain the sequential biases observed in perceptual judgments (Saarela et al., 2023). Serial dependence and CTB magnitudes also do not correlate with each other in numerosity or speed perception, suggesting at least partial independence of the effects (Burgio, Moscoso, Anobile, & Castaldi, 2025; Tonelli, Burr, & Alais, 2026). A dissociation between CTB and serial dependence has also been observed in two clinical populations: individuals with autism spectrum disorder show CTB comparable with neurotypical individuals but reduced serial dependence, whereas those with dyslexia exhibit diminished CTB despite having serial dependence comparable with neurotypical individuals (Lieder et al., 2019). Furthermore, in orientation perception, serial dependence is associated with alpha-band perceptual oscillations, whereas CTB does not exhibit such oscillations, indicating distinct neural mechanisms for short- and long-term history effects (Xie, Burr, & Morrone, 2025). However, CTB and cardinal bias were indistinguishable in this study. In contrast, modeling approaches have indicated that serial dependence and CTB arise from a shared mechanism (Boboeva, Pezzotta, Clopath, & Akrami, 2024; Glasauer & Shi, 2022; Tong & Dubé, 2022; Wang et al., 2023) or that CTB is a statistical artifact instead of an independent effect (Boboeva et al., 2024; Sailor & Antoine, 2005). Both effects can be described with Bayesian observer models that differ in the way that the prior distribution is updated over the course of the experiment. The different ways of updating the prior can be conceptualized as differences in beliefs about the stimulus generation: CTB emerges when an observer assumes a stationary stimulus distribution, whereas updating the prior after each trial leads to serial dependence (Glasauer & Shi, 2022).

All previous studies have used indirect methods to study these two effects. We take advantage of the circularity of orientation space to directly study the independence of the sequential effects in different time scales. CTB cannot exist for circular stimulus spaces, such as orientation, because a mean cannot be computed for a circular uniform distribution. CTB can, however, theoretically be observed when only a section of a circular stimulus space is used, for example only orientations between 0° and 45°, because in this case the distribution has a mean. In the case of hue, which is another circular stimulus space, CTB emerges with a restricted range of hues (Olkkonen et al., 2014). We use these two conditions to separate CTB from cardinal bias. The same conditions can also be used to separate CTB and serial dependence. As serial dependence in orientation judgments has been widely observed (e.g., Fischer & Whitney, 2014; Fritsche, Mostert, & de Lange, 2017; Pascucci et al., 2019), it appears that CTB is not a prerequisite for serial dependence. However, CTB could still arise from the accumulation of serial dependence, or they could have shared mechanisms. We examine this by comparing serial dependence in the two conditions.

## Experiment 1

### Methods

#### Participants

Twenty-four participants (17 women, 5 men, 2 non-binary) with mean age of 22.4 years (range, 19–32 years) took part in experiment 1. All participants had normal or corrected-to-normal visual acuity in both eyes (self-reported). Participants were given an opportunity to listen to instrumental music of their choice on low volume to improve engagement (Homann, Drody, & Smilek, 2023); six participants chose to do so. There was no evidence for differences in response times, absolute errors, CTB slopes, or serial dependence amplitudes between participants who listened or did not listen to music.

Participants gave written informed consent before participation and had the right to withdraw from the experiment at any point. The University of Helsinki Research Ethics Committee in the Humanities and Social and Behavioural Sciences reviewed the experimental protocol and concluded that the study follows the ethical principles of research in the humanities and social and behavioral sciences issued by the Finnish Advisory Board on Research Integrity. Participants were recruited at the University of Helsinki and, as a compensation for their time, received either a gift card worth 10€ or course credit.

#### Apparatus

The experiment was run on an HP Z230 Desktop PC with a 23-inch ViewPixx monitor (1,920 × 1,080 px, 100 Hz). The maximum luminance of the screen was 250 cd/m^2^. The viewing distance was held constant at 100 cm with a chin rest, and the experiment was completed in a darkened room. Participants gave their responses via a wired computer mouse. The experiments were implemented in Matlab (R2016b) with PsychToolbox (Brainard, 1997; Kleiner, Brainard, & Pelli, 2007; Pelli, 1997).

#### Stimuli

Stimuli were low-contrast Gabor patches presented centrally on a uniform gray background (125 cd/m^2^). The Gabor patches had a Michelson contrast of 0.25. The patches consisted of a sine-wave grating, with a spatial frequency of 0.5 cycles per degree of visual angle and a phase of 0° or 180°, presented in a Gaussian envelope with a standard deviation of 1 degree of visual angle.

Stimuli were backward masked to minimize negative afterimages. The mask consisted of white noise filtered with a Gaussian bandpass filter with the same peak frequency as the Gabors, 0.5 cycles per degree of visual angle, and 1 octave full width at half maximum. The mask was also windowed in a Gaussian envelope with a standard deviation of 1 degree of visual angle. The peak contrast of the mask was adjusted to be 0.40 (Michelson).

The response probe was a white (250 cd/m^2^) 0.5 degree of visual angle wide line windowed with a 1 degree of visual angle Gaussian envelope.

#### Procedure

Participants completed two conditions in a blocked design: the full circle condition and the section condition (Figure 1b). In the full circle condition, Gabor patch orientations were drawn at random from 36 orientations ranging between 0° and 175° with a step size of 5°. In the section condition, orientations were sampled from 12 orientations from a 70° wide section of the full circle with a step size of 5°. The section was centered on one of four orientations: vertical cardinal axis (0°), horizontal cardinal axis (90°), clockwise diagonal (45°), or counterclockwise diagonal (135°), counterbalanced between participants.

**Figure 1.**
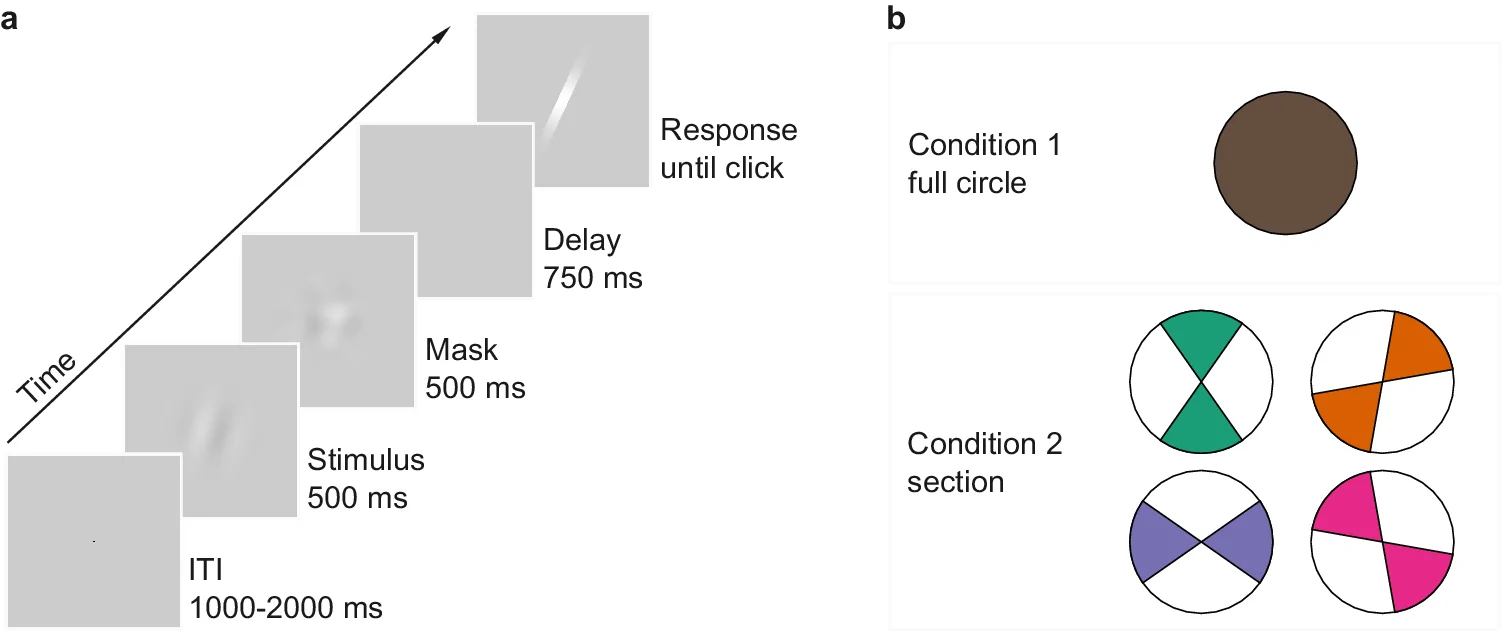
(**a**) The sequence of events in one trial in experiment 1. (**b**) Illustration of the two conditions in the study. In the full circle condition, orientations were uniformly distributed along the whole circle. In the section condition, a 70°-wide section was used. Each participant was assigned one of the four different section options, with six participants per section.

Participants completed both conditions during a 1-hour session. Half of the participants completed the full circle condition first, and the other half completed the section condition first. Both conditions consisted of 260 trials each. Participants could take a short break every 65 trials and a longer break between the two conditions. Participants were not informed that the two conditions were different before they had completed both conditions. After the experiment, when asked, 12 of the 24 participants reported to not have noticed any difference between the two conditions.

Each trial started with the presentation of a fixation dot in the center of the screen for 1 to 2 seconds (sampled randomly from a uniform distribution) (Figure 1a). Participants were instructed to keep their gaze at the center of the screen for the duration of the trial. Next, a Gabor patch was presented for 500 ms. Immediately after it, a mask was presented for 500 ms. After a 750-ms delay, the response probe appeared at the center of the screen with a random orientation. Participants then adjusted the orientation of the probe using a computer mouse by moving the mouse left or right to turn the response probe counterclockwise or clockwise, respectively. When participants were satisfied with the orientation, they clicked the left mouse button. This started the next trial.

Before starting the actual experimental blocks, participants completed 15 practice trials to familiarize themselves with the adjustment procedure. They also received feedback on these trials: after clicking, a white and a black line were presented centrally along with an emoticon below them. The orientation of the white line corresponded with the participant’s response and the black line had the same orientation as the stimulus. If the absolute response error was less than 13°, the emoticon was happy—:)—and if the error was larger than 13°, it was sad—:(—. The practice trials were not included in the analysis.

#### Analysis

In accordance with standard practices in the serial dependence literature (Pascucci et al., 2023), we removed trials with response times below 400 ms or exceeding each participant's mean response time by more than 3.5 standard deviations. We also removed trials in which response errors were more than 3 standard deviations away from the mean response error of each participant. Of the trials, 1.36% were removed in the full circle condition, and 1.27% were removed in the section condition. This resulted in a total of 12, 316 trials (1.31% removed). For analysis of serial dependence, we also removed the first trial after each break because in these trials there was no preceding trial or the time between preceding and current trials was long.

Uncorrected response errors were calculated as the circular difference between the response orientation and the orientation of the current stimulus in each trial. Because cardinal biases can cause spurious serial dependence (Pascucci et al., 2019) and cardinal biases can mask CTB or other stimulus distribution-specific effects, we the calculated cardinal bias residual errors using the full circle condition. For each participant separately, we fit a Fourier series expansion truncated at the fourth harmonic to the response errors in the full circle condition as a function of the stimulus and removed the resulting predicted mean errors from the response errors in both conditions. Because there were further systematic error patterns in the section condition, to remove those in the analysis of serial dependence, we calculated condition-specific residual errors by the same procedure for each participant and each condition separately. Cardinal bias residual errors and condition-specific errors were then equal in the full circle condition.

Serial dependence was quantified using a derivative of the von Mises density function. This function is better suited for quantifying serial dependence in circular stimulus spaces than the often-used derivative of Gaussian function because it accounts for circularity. The von Mises distribution is a probability distribution in circular space that is analogous to the normal distribution, which is a special case of a Gaussian function in linear space. A few previous studies have used a derivative of the von Mises density function to estimate serial dependence (Kondo, Murai, & Whitney, 2022; Little & Clifford, 2025; Manassi et al., 2021; Trübutschek & Melloni, 2023).

We derived a new, differently scaled version of the derivative of von Mises (DovM) function (Equation 1). This DovM function has the same feature as the derivative of Gaussian function, where the parameter α given by fitting the curve matches the actual maximum half-amplitude of the curve. The function is of the form

(1) $$\begin{array}{c} DovM\left(x|\alpha ,\kappa \right)=g\left(\kappa \right)\alpha \kappa \sin\left(x\right)e^{\kappa \cos(x),}\; \end{array}$$

where *x* is the difference between the preceding and the current stimulus in radians such that the largest possible difference, for example, 90° for orientation, maps to ±π. In other words, ±π is the stimulus difference at which serial dependence must be zero for the model not to contradict itself. The parameter α is the half-amplitude and κ is the measure of concentration. Finally, *g*(κ) is a scaling function such that α matches the maximum of the curve. See Supplementary materials A for more information on the function.

When fitting the curve, instead of the parameter κ, we fit the angular difference where the DovM curve has its maximum value, the peak location (θ), because it is more interpretable. The peak location θ and the concentration κ are connected such that

(2) $$\begin{array}{c} \kappa =\frac{\cos(\theta )}{\sin^{2}\left(\theta \right)}\; \end{array}$$

We set the lower and upper bounds for the peak location at 5° and 45°, respectively. The DovM curves were fit using least-squares with the *fmincon* function in Matlab. The goodness of the fits was visually estimated by plotting the curves. In cases where a participant had both attractive and repulsive effects, the curve was fit to the more narrowly tuned attractive component, because this bias is generally described as serial dependence (Pascucci et al., 2023). As a sanity check, we fit the same curves to data where the stimulus order had been shuffled. This removed any statistically significant serial dependence from the data.

Data analysis was done in Matlab (R2024a) except for Bayes factors calculations, which were done in R (version 4.4.2). Bayes factors were computed with the package BayesFactor (version 0.9.12-4.7) (Morey & Rouder, 2024) using the uninformative Jeffreys prior for population variance and the standard Cauchy prior for the effect size.

### Results

Participants, on average, had a bias away from cardinal orientations in the full circle condition (Figure 2a). To quantify this bias, we binned trials into two groups based on whether the closest cardinal axis was clockwise or counterclockwise from the presented stimulus. For each participant, we calculated the difference in the mean response error between these bins. In the full circle condition, the mean difference in response errors was −5.39, indicating a bias away from the cardinal axes, *t*(23) = −5.06, *p* < 0.001, *BF10* = 616. In the section condition, the mean difference was similar at −5.53, *t*(23) = −3.16, *p* = 0.004, *BF10* = 9.70, but the overall pattern of errors differed from that observed in the full circle condition (Figure 2b).

**Figure 2.**
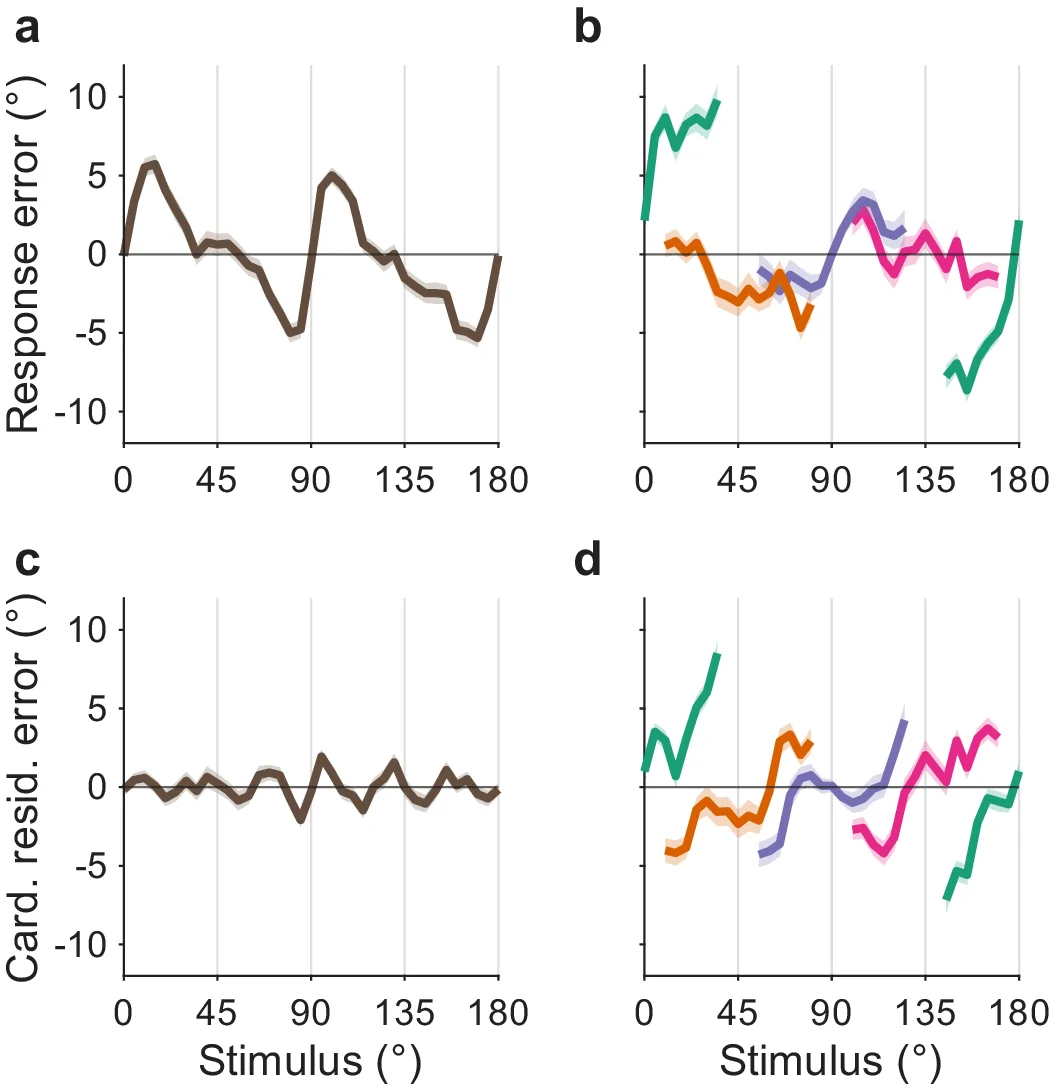
Long- and medium-term history biases in experiment 1. (**a**) The participants had a clear bias away from the cardinal directions in the full circle condition. The figure depicts pooled data across all participants. The line shows the mean response error and the shaded area shows ± standard error. (**b**) In the section condition, the pattern of biases was altered such that in some sections (vertical, green) participants seemed to have more bias than the full circle condition but in others (diagonals, orange and pink) there was less bias. Each colored line represents data from the six participants who completed that section. (**c**) When each participant’s individual cardinal biases were removed from the response errors, the systematic biases were removed from the full circle condition. (**d**) When the errors explained by cardinal bias were removed in the section condition, another systematic effect was revealed. The residual errors in the section condition had a positive slope. This is contrary to what would be observed in the case of CTB.

To analyze the CTB and serial dependence independently of cardinal biases, we estimated the cardinal biases of each participant separately by fitting a Fourier series expansion truncated at the fourth harmonic to the response errors in the full circle condition. Cardinal bias residual errors reflect the residual response error without cardinal biases, assuming that the cardinal biases are identical in the two conditions. CTB was quantified as the slope between stimulus and cardinal bias residual error. If participants had a CTB, the slopes would be negative, as responses to stimuli counterclockwise from the center of the section would be biased toward the center and vice versa. Conversely, the participants had positive slopes, *M* = 0.12, *t*(23) = 5.32, *p* < 0.001, *BF10* = 1,109. That is, they had a bias away from the center of the section, independent of cardinal biases (Figure 2c). There were no statistically significant differences in the slopes between different sections, *F*(3,20) = 1.22, *p* = 0.330, or between the participants who completed the full circle condition first and the participants who completed the section condition first, *t*(22) = 1.81, *p* = 0.251, *BF10* = 0.616. There were also no statistically significant differences between those participants who noticed a difference between the full circle and section conditions and those who did not, *t*(22) = −0.58, *p* = 0.565, *BF10* = 0.423. These factors are therefore unlikely to explain the results.

The positive slopes could also be a statistical effect of repulsive serial biases. To control for them, we removed the shared variance of the previous stimulus and the current stimulus by orthogonalizing the stimulus vector with respect to the difference between current and preceding stimuli using the Gram–Schmidt process (Supplementary Materials B). If the positive slopes for stimulus errors as a function of the current stimulus are driven by a repulsive serial bias, this orthogonalization makes the slopes zero; see Supplementary Materials B for a simulation showing that this happens. However, the slopes remained positive with a mean of 0.11, *t*(23) = 5.32, *p* < 0.001, *BF10* = 1108.

To analyze one-back serial dependence in both conditions, we fit the DovM curves to each participant’s data separately. In the full circle condition, the mean half-amplitude was 1.38°, *t*(23) = 3.14, *p* = 0.005, *BF10* = 9.35, and the mean peak was at 17.09°, which means that, on average, participants had attractive serial dependence. Similarly in the section condition, the mean half-amplitude was 1.33°, *t*(23) = 4.19, *p* < 0.001, *BF10* = 88.1, and the peak was at 10.66°. Figure 3a depicts fits to the pooled data. The serial dependence curve in the full circle condition seems to be wider than the curve in the section condition. Comparing the two conditions within subject, there was no evidence for a difference between the half-amplitudes in the two conditions, *M* = 0.05, *t*(23) = 0.11, *p* = 0.916, *BF10* = 0.216. The peaks, however, were different between the two conditions, such that the curves were more narrow in the section condition, *M* = 6.42, *t*(23) = 2.93, *p* = 0.008, *BF10* = 6.12.

**Figure 3.**
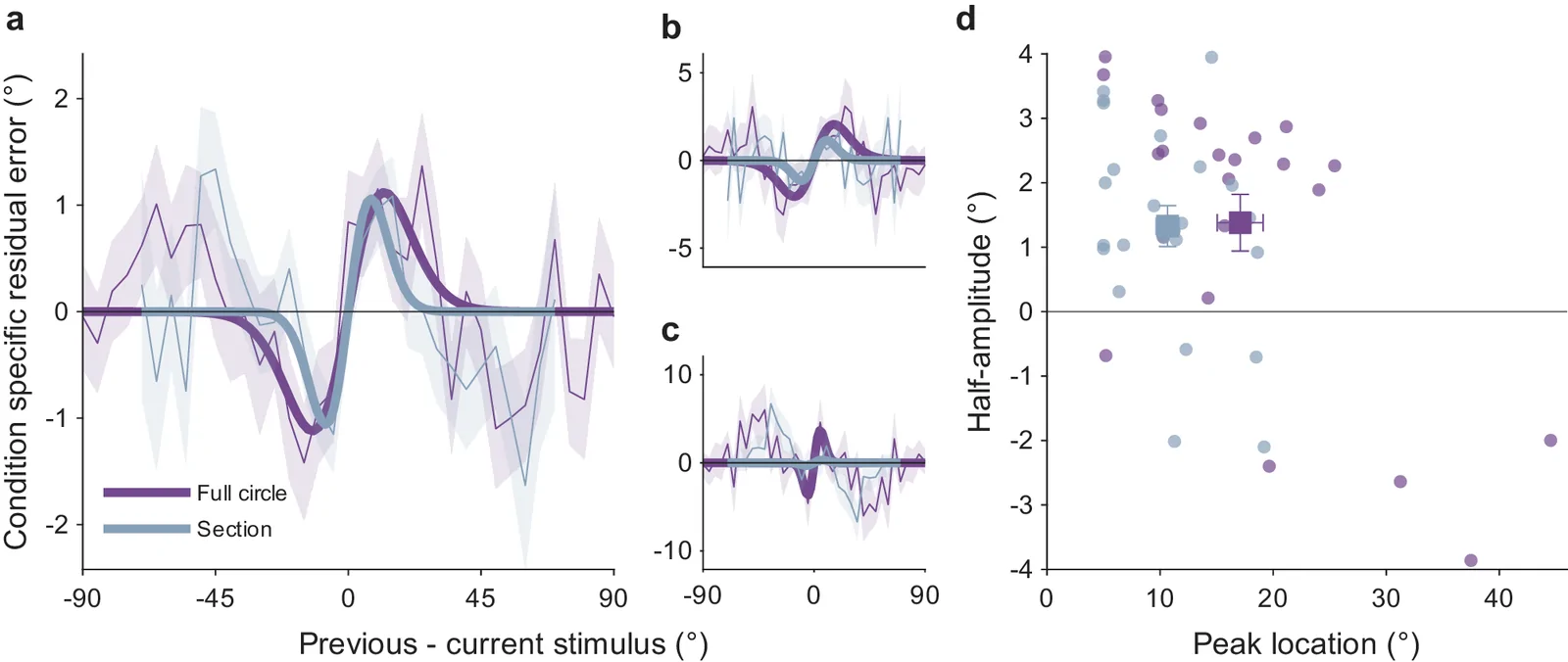
Analysis of serial dependence between the two conditions. (**a**) DovM curves fit to the pooled data separately for the two conditions. The thin lines are the mean condition-specific response errors and the shaded areas are their standard errors. (**b**) An example of curve fits for one participant. This participant had clear attractive serial dependence when the difference between consecutive stimuli was small, but they had a repulsive serial bias when the difference was larger, especially in the full circle condition. (**c**) Another example participant with a large repulsive serial bias when the difference between previous and current stimulus was large but also a narrowly tuned attractive serial dependence. (**d**) Half-amplitudes and peaks of the individual fits plotted against each other. The squares represent the mean half-amplitudes and peaks for the two conditions, and error bars are the standard errors.

Many participants who exhibited attractive serial dependence also showed a repulsive bias when the difference between the current and preceding stimuli was greater (e.g., the participant in Figure 3b). This can cause the DovM (or derivative of Gaussian) curve fit to be narrower than the actual response error curve. As the stimulus difference increases, the fitted curve tends to approach zero quickly to minimize errors introduced by the negative dips. To make sure that the difference in peak location was not the result of larger repulsive serial biases in the section condition, we fit a sum of two DovM functions to each participants’ data (Figure 4). One of the components was to capture the more narrowly tuned attractive effect and the other to capture the repulsive serial bias for larger orientation differences. Therefore, the fitting procedure was restricted so that the attractive component had to have a positive half-amplitude and the repulsive component had to have a negative half-amplitude. Furthermore, the peak location of the attractive component was to be closer to zero than the peak location of the repulsive component. From these curve fits, we extracted the maxima and minima and their locations from the open interval between 0° and 90° using numerical differentiation. For participants who did not have a defined maxima or minima, the value 0 was used instead in *t*-tests.

**Figure 4.**
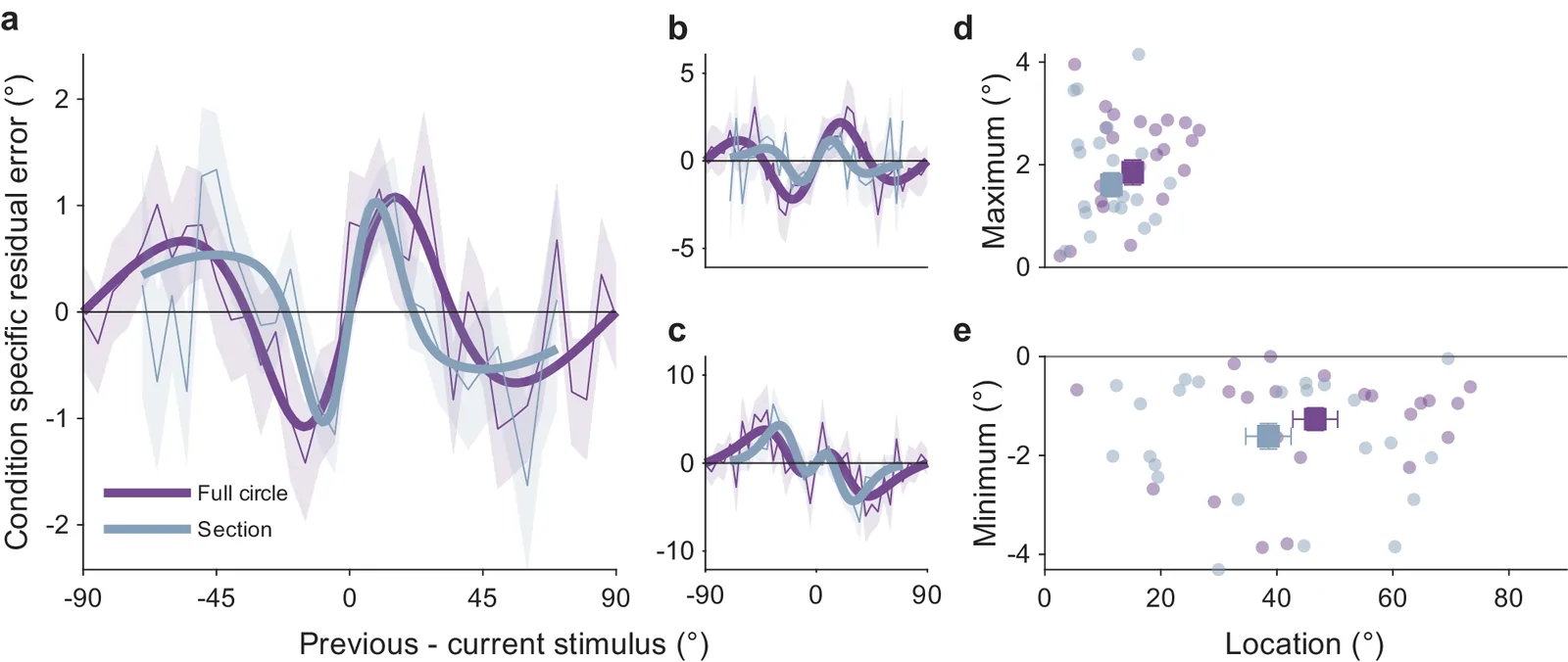
Analysis of serial dependence using the sum of two DovM functions. (**a–c**) This function was able to capture the repulsive biases for large orientation differences. (**d, e**) Numerically derived maxima and minima of the individual participants’ curves plotted as a function of the orientation difference, where the maximum or minimum bias was. Squares show the mean values. Participants who had no maxima or minima in a given condition have not been plotted.

The mean maximum derived by the sum of two DovM functions was 1.85° and 1.61° in the full circle and section conditions, respectively. This difference in half-amplitudes was not significant, *t*(23) = 0.76, *p* = 0.457, *BF10* = 0.278. The attractive serial dependence was more narrowly tuned in the section condition: the maxima were located at 15.25° and 11.48° in the full circle and section conditions, respectively, *t*(19) = 5.44, *p* < 0.001, *BF10* = 814. Similarly, there was no evidence for a difference between the minima in the two conditions with a mean minimum in the full circle condition being −1.27 and in the section condition −1.61, *t*(23) = 1.19, *p* = 0.245, *BF10* = 0.404, but there was a difference in the locations of minima, *t*(21) = 3.74, *p* = 0.001, *BF10* = 30.3, such that the minima was closer to zero in the section condition (*M* = 38.55°) than in the full circle condition (*M* = 46.62°).

## Experiment 2

We did not find a CTB despite an attractive serial dependence in experiment 1. The magnitude of CTB is dependent on internal and external noise, such that more noise results in a stronger CTB and less noise can flip the effect to be repulsive (Olkkonen et al., 2014). It is possible that the stimuli had too high a contrast or the response delay was too short to cause enough noise, that is, uncertainty about the stimulus for CTB to appear. In addition, uncertainty related to the stimulus also increases serial dependence magnitude (Bliss et al., 2017; Ceylan, Herzog, & Pascucci, 2021; Cicchini, Mikellidou, & Burr, 2018; Gallagher & Benton, 2022). Increasing noise should then make both attractive effects stronger. For this reason in experiment 2 we repeated the same experiment with small adjustments to make the task more difficult. The noise manipulation was successful: standard deviations of response errors were larger in experiment 2 compared with experiment 1, *t*(46) = −2.34, *p* = 0.024, Cohen’s *d* = −0.66.

### Methods

For experiment 2, 24 new participants (13 women, 10 men, 1 non-binary) were recruited. The mean age of the participants was 27.2 years (range 19–57 years). One participant was excluded for having a response error standard deviation that was approximately twice as large as the average response error standard deviation of other participants (23.89° vs. 11.98°), and another participant was recruited to replace the excluded participant. The requirements to participate were the same as in experiment 1. In experiment 2, participants were not offered the chance to listen to music because so few participants chose to do so in experiment 1.

The methods of experiment 2 were almost identical to experiment 1 (Figure 5). The contrast of Gabor patches was lowered to 0.10 and the presentation time shortened to 250 ms. Correspondingly, the delay between the mask and the response probe was lengthened to 1,000 ms so that trial length was the same in the two experiments. The analysis was conducted with the same pipeline as experiment 1. In experiment 2, 2.40% of trials in the full circle condition and 2.20% of trials in the section condition were removed during preprocessing, giving a total of 12,193 trials (2.30% removed).

**Figure 5.**
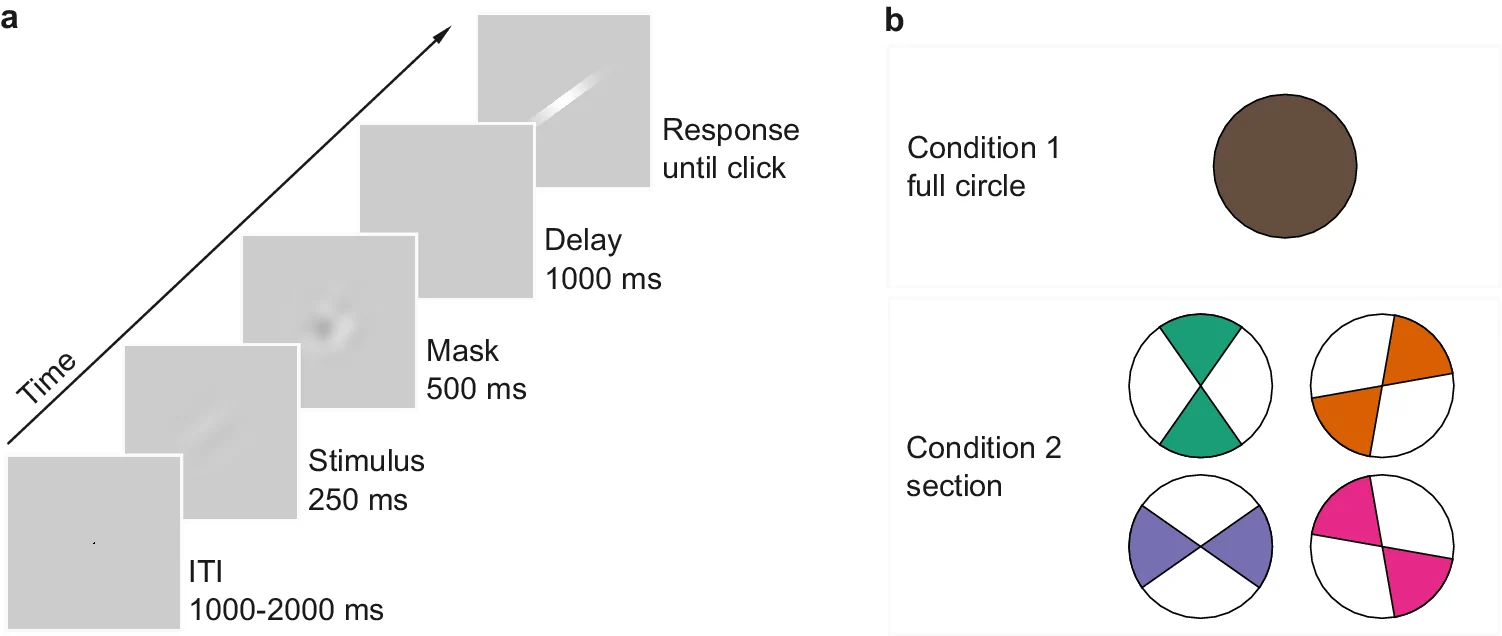
(**a**) The procedure in experiment 2 was almost identical to that in experiment 1. (**b**) The same conditions and sections were used in experiment 1 as in experiment 2.

### Results

Participants in experiment 2 also had a cardinal bias (Figure 6a, 6b). The mean response errors differed across participants depending on whether the closest cardinal axis was clockwise or counterclockwise compared with the current stimulus orientation in the full circle condition, *t*(23) = −6.63, *p* < 0.001, *BF10* > 10^4^, and in the section condition, *t*(23) = −3.85, *p* < 0.001, *BF10* = 41.6.

**Figure 6.**
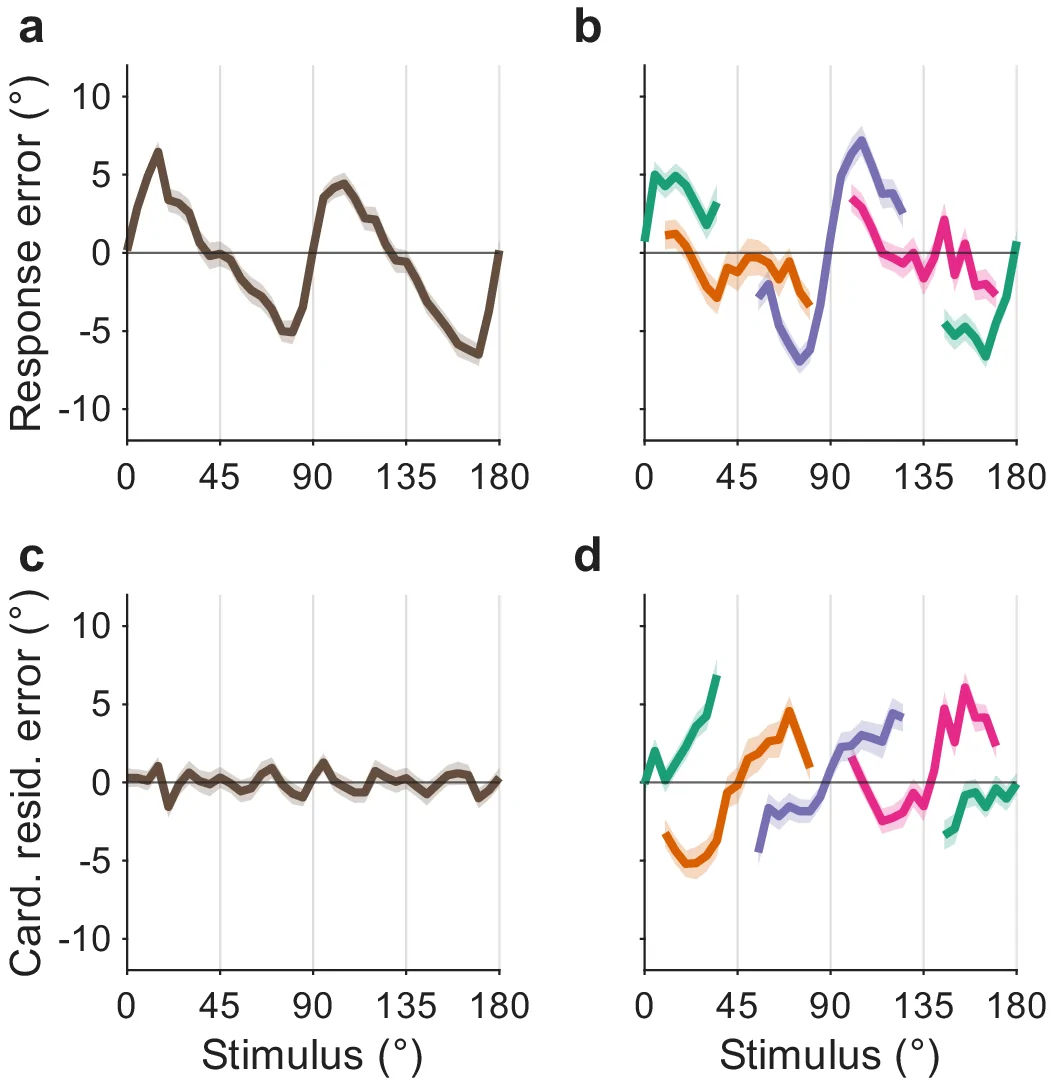
Long-term and medium-term biases in experiment 2. All figures show data pooled across participants. The thick lines show the mean response errors or mean residual response errors and the shaded area shows the standard error. (**a**) On average, participants showed a bias away from cardinal orientations (0° and 90°) in the full circle condition. (**b**) Biases in the section condition before controlling for cardinal bias. (**c**) Systematic cardinal biases were controlled for in the full circle condition. (**d**) When cardinal bias was controlled for in the section condition, participants in all sections showed a bias away from the center of the section.

We repeated the same analysis for CTB as in experiment 1. Despite greater uncertainty about the stimulus orientation, the participants showed a repulsive bias away from the center of the section (Figure 6c). The mean slope of the bias was 0.11, *t*(23) = 5.55, *p* < 0.001, *BF10* = 1817. The slopes stayed positive and highly statistically significant also after controlling for repulsive biases from the preceding stimulus, *M* = 0.11, *t*(23) = 5.06, *p* < 0.001, *BF10* = 611. There were no statistically significant differences between the sections, *F*(3,20) = 0.23, *p* = 0.876. The participants who completed the full circle condition first had a larger repulsive bias than the participants who completed the section condition first, difference in means 0.087, t(22) = 2.31, *p* = 0.030, BF10 = 2.340, but the evidence for the difference was weak and the full circle condition group still had a positive slope (*M* = 0.07).

Lastly, we analyzed serial dependence independent of cardinal biases and the bias away from the center of the section. Participants had on average attractive serial dependence in both conditions (Figure 7). When quantified with one DovM curve, the maximum half-amplitude of serial dependence was 1.42° in the full circle condition, *t*(23) = 2.72, *p* = 0.012, *BF10* = 4.07, and 1.29° in the section condition, *t*(23) = 4.08, *p* < 0.001, *BF10* = 68.6, (Figure 7b). There were no differences in the half-amplitudes between the conditions, *t*(23) = 0.27, *p* = 0.789, *BF10* = 0.222. The mean peaks of the curves were at 15.71° and 12.74°. Unlike in experiment 1, there was no evidence for a difference in the peak locations of the curves, *t*(23) = 1.18, *p* = 0.251, *BF10* = 0.398. A more detailed analysis using a sum of the two DovM curves revealed that the maximum half-amplitude of the attractive serial dependence component was larger in the full circle condition than in the section condition, *M* = 0.76, *t*(23) = 2.64, *p* = 0.015, *BF10* = 3.51, but there was no difference in its location, *t*(21) = −0.03, *p* = 0.975, *BF10* = 0.223, or in the half-amplitude of the repulsive serial dependence component, *t*(23) = 0.66, *p* = 0.517, *BF10* = 0.261. The evidence about the locations of the repulsive components between the conditions was inconclusive, *t*(18) = −1.06, *p* = 0.303, *BF10* = 0.389.

**Figure 7.**
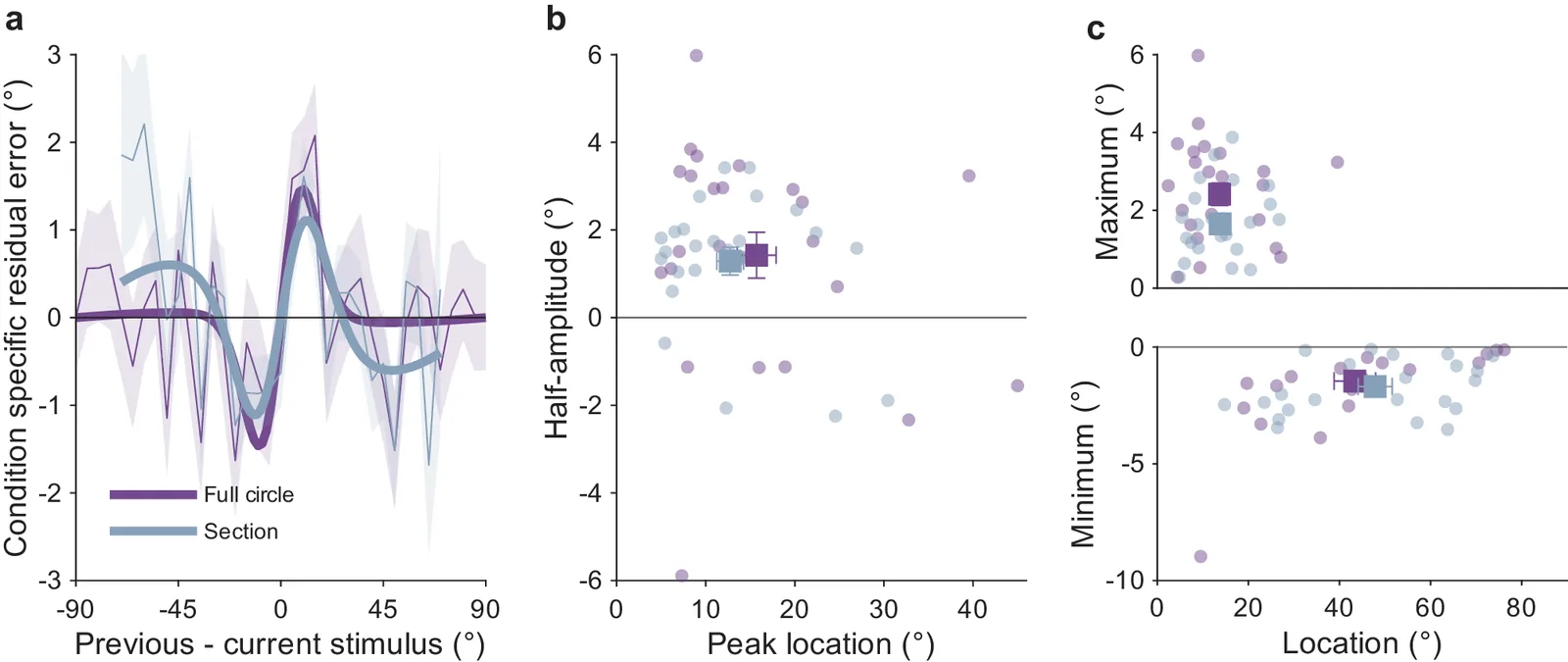
Serial dependence in experiment 2. (**a**) The sum of two DovM curves fit to the pooled data. (**b**) Half-amplitudes and peak locations from DovM fits to individual participants. The mean half-amplitude and peak location do not seem to differ between the two conditions. (**c**) Numerically derived maxima, minima, and their locations from the sum of two DovM curves. The maxima seem to be larger in the full circle condition, meaning that attractive serial dependence had a greater magnitude in that condition compared with the section condition.

## Discussion

We examined in two experiments the concurrent existence of short-, medium-, and long-term sequential effects in orientation judgments when stimulus orientations were drawn from the full circle or from a section of all orientations. Participants exhibited a bias away from cardinal directions in line with previous literature (Bae, 2022; de Gardelle et al., 2010) in both conditions and both experiments. When stimulus orientations were drawn from a section of all orientations, against expectations, participants did not have CTB but a bias away from the center of the section. Participants did, however, have attractive serial dependence in both conditions and both experiments on average, although there was variation between individuals. In the experiment with higher contrast stimuli, serial dependence was equivalent in magnitude in the two conditions but was more narrowly tuned in the section condition. The results were slightly different in the second experiment using lower contrast stimuli: serial dependence did not differ much between the two conditions.

Instead of CTB, participants exhibited a bias away from the center of the stimulus distribution. Previously, CTB has been observed using many different stimulus types in vision, including sections of circular distributions in the case of hue (Olkkonen et al., 2014). In addition, it emerges in other sensory modalities (Raviv et al., 2012; Riskey, Parducci, & Beauchamp, 1979; Tonelli, Phan, & Alais, 2025), indicating its generality as a psychological phenomenon. In Bayesian observer models, CTB is often considered a natural consequence of observers learning the statistical properties of the stimulus distribution and using it to make inferences that are optimal under uncertainty (Jazayeri & Shadlen, 2010). Human observers have been shown to behave in a way that suggests that they are able to infer even relatively nontrivial stimulus distributions and use them as priors (Acerbi, Wolpert, & Vijayakumar, 2012). Additionally, humans can estimate means in circular stimulus spaces, such as motion direction (Hubert-Wallander & Boynton, 2015). In motion direction perception, a bias toward the most common motion orientations arises rapidly after only a few minutes of task performance (Chalk, Seitz, & Series, 2010). In the present study also, the majority of participants could identify that one of the conditions only used a section of orientations. Given the established understanding of CTB in other stimulus types and modalities, the repulsive bias away from the center of the stimulus distribution in orientation judgments is unexpected.

A repulsive effect like this could arise from adaptation. Adaptation is often defined as a short-term effect operating on a timescale of seconds to a few minutes, but there are also adaptation effects that operate on different, longer timescales (Bao & Engel, 2012; Kording, Tenenbaum, & Shadmehr, 2007; Vul, Krizay, & MacLeod, 2008). One proposed function of adaptation is to allow better discrimination of stimuli that are common or that we are more exposed to (Attneave, 1954; Barlow, 1961; Wark, Lundstrom, & Fairhall, 2007; Webster, 2015). Within the efficient coding framework, adaptation reflects the reallocation of neural resources to match the statistical structure of the environment (Laughlin, 1981; Simoncelli & Olshausen, 2001; Wei & Stocker, 2015). One classical mechanism for this is decorrelation: reducing redundancy among population activity patterns so that common or predictable stimulus features are encoded more independently (Barlow & Földiák, 1989; Mattar, Olkkonen, Epstein, & Aguirre, 2018). Such efficient coding processes improve the discriminability of common stimuli but also introduce repulsive biases away from them. Bayesian efficient coding models formalize this by combining efficient encoding with Bayesian decoding, allowing them to explain both attractive and repulsive perceptual biases (Mao, Rothkopf, & Stocker, 2025; Wei & Stocker, 2015). In the context of sequential effects, these Bayesian efficient coding models have been shown to predict concurrent attractive and repulsive short-term serial effects (Fritsche, Spaak, & de Lange, 2020). An interesting future question is why studies using other stimulus types than orientation find an attractive CTB and why orientation causes repulsive medium-term adaptation effects found in the present study.

Another possible explanation for the bias away from the center of the stimulus distribution is that it is a statistical artifact of the short-term repulsive serial biases that emerge when the difference between the current and the preceding stimulus is large. The difference between the preceding and the current stimulus and the difference between the mean of the whole stimulus distribution and the current stimulus are highly correlated (Glasauer & Shi, 2022; Saarela et al., 2023; Tong & Dubé, 2022). Consequently, the repulsive serial bias can appear as a bias away from center when analyzed as a function of the current stimulus value. However, our analysis suggests that the bias was not a statistical artifact because it persisted even after accounting for the response errors related to the previous stimulus. This means that the bias is probably not driven just by the preceding stimulus. Previously it has been shown that both a short-term repulsive aftereffect from the immediate past and a longer-term repulsive effect caused by stimulus history several minutes in the past can be identified (Gekas & Mamassian, 2025; Gekas, McDermott, & Mamassian, 2019). The repulsive effect observed here thus more likely corresponds with the latter one. A second artifactual possibility is that cardinal bias was not the same in the full circle and section conditions. In this case, the repulsive bias in the section condition could actually reflect a change in cardinal bias. However, this explanation is incompatible with the common explanation of cardinal bias as a low-level and long-term effect (Appelle, 1972; Li et al., 2003). In experiment 1, there were also no differences in the repulsive bias depending on which condition was done first. However, there was weak evidence for a difference in experiment 2, so this explanation cannot be fully ruled out.

The repulsive bias away from the center also appears to be in contrast with existing models on serial dependence and CTB, most of which do not include a separate mechanism for CTB but predict that CTB emerges from the existence of serial dependence (Boboeva et al., 2024; Tong & Dubé, 2022; Wang et al., 2023) or that they arise from a shared mechanism (Glasauer & Shi, 2022). Here, however, we observed no CTB, although participants did have attractive serial dependence. The existence of attractive serial dependence is therefore not a sufficient condition for CTB, and serial dependence does not necessarily build up to CTB. It is, however, worth noting that these models were constructed in the context of linear stimulus spaces and most of them predict that serial dependence is linear as a function of the previous stimulus, without feature tuning. According to a meta-analysis, serial dependence exhibits feature tuning in many different stimulus types such that the bias is maximal when the difference between current and preceding stimuli is 20% to 30% of the whole stimulus space (Manassi et al., 2023). However, all of the studies in the meta-analysis used circular stimulus spaces, either naturally circular such as orientation or motion direction in two dimensions, or made circular by morphing, such as shapes and faces. In linear stimulus spaces, serial dependence can appear to be linear or near linear: for example, serial dependence in estimates of visual variance (Suárez-Pinilla et al., 2018), numerosity (Fornaciai & Park, 2020), line length (Saarela et al., 2023) or time interval length (Glasauer & Shi, 2022) seem to have less or no feature tuning. This could partially be explained by the range of stimuli; if the range used in the experiment is too narrow, there will not be any trials where the difference between consecutive stimuli is large enough to capture the ends of the tuning curves and the possible peripheral repulsive effects (Manassi et al., 2023). Additionally, it could be that feature tuning is a feature of serial dependence mainly in circular stimulus distributions, as they do not allow for linear serial dependence. Applying linear serial dependence in a circular stimulus space would lead to a contradiction: it would cause concurrently repulsive and attractive biases at maximum stimulus differences (e.g., 90° for line orientation). If feature tuning is different in linear and circular stimulus spaces, serial dependence has to be modeled differently in these spaces.

The final insight into CTB in orientation perception comes from the category adjustment model (CAM) (Huttenlocher et al., 2000). The model proposes that CTB arises from the integration of category-level information with uncertain sensory inputs (Huttenlocher et al., 2000). According to the category adjustment model, during a typical experiment, the observer learns the stimulus distribution that forms a new perceptual category. This new category is then used to reduce uncertainty. In orientation reproduction, observers often have a bias away from the cardinal orientations and toward the oblique ones, and this bias is explained by better discriminability of near-cardinal orientations (Appelle, 1972; de Gardelle et al., 2010). However, from the category adjustment model perspective, this cardinal bias could be interpreted as a consequence of observers dividing orientations into distinct categories. If the categories are formed such that cardinal orientations act as the boundaries of these categories, observers will exhibit a bias away from the cardinal orientations and toward the center of the category. If these perceptual categories are strongly established before the experiment, 260 trials may be insufficient to change them to align with the new stimulus distribution, leading to no apparent CTB independent of cardinal bias. However, from the point of view of the category adjustment model, cardinal bias and CTB are one and the same. Categorical perception of orientations has been proposed before (Balikou et al., 2015; Durgin & Li, 2011; Mao & Stocker, 2024; Quinn, 2004; Wolfe, Friedman-Hill, Stewart, & O’Connell, 1992). Still, we do not find this explanation fully satisfactory because, in other stimulus contexts, CTB is a medium-timescale effect arising from the current stimulus distribution, rather than from long-term categories. Hue judgments also exhibit biases due to long-term color categories (Bae, Olkkonen, Allred, & Flombaum, 2015), but additionally, independently of them, CTB caused by the current stimulus distribution can be observed (Olkkonen et al., 2014). It is perhaps natural to assume that orientation and hue should exhibit similar biases because both variables are categorical and circular, but this may be a naive assumption. The perceptual geometry of orientation space has a straightforward interpretation, whereas we do not have a good understanding of the perceptual geometry of color space. That we find such different patterns of biases for orientation and hue is perhaps added proof of their fundamental difference.

Lastly, this study examined serial dependence in orientation judgments when orientations were sampled from a circular uniform distribution and a more narrow section of orientations. In the first high-contrast stimulus experiment, the feature tuning width of serial dependence was altered by the shape of the stimulus distribution. Specifically, when stimuli were drawn from a narrower distribution, serial dependence feature tuning was also narrower compared with when stimuli were drawn from the full circular distribution. This result is aligned with theories about the function of serial dependence and the concurrent repulsive serial bias. In the real, autocorrelated world, serial dependence is considered to be beneficial in reducing uncertainty and errors caused by noise and in this way stabilizing perception (Fischer & Whitney, 2014; Kiyonaga, Scimeca, Bliss, & Whitney, 2017). Conversely, the repulsive bias for larger feature differences aids in discriminating stimuli that are truly different (Fritsche et al., 2017; Fritsche et al., 2020; Pascucci & Plomp, 2021; Pascucci et al., 2019). These two biases work together to balance stability and discriminability (Fritsche et al., 2017; Moon & Kwon, 2022; Pascucci et al., 2019; Park, 2025; Sadil, Cowell, & Huber, 2024). Interestingly, the temporal statistics of stimuli alter serial dependence, which challenges the idea that serial dependence arises from a single stability prior (Blondé, Kristjánsson, & Pascucci, 2023) and suggests a dynamic mechanism of attractive and repulsive serial biases. This idea is also supported by experiment 1 of the current study. When the stimulus distribution became more narrow, as it was in the section condition, smaller stimulus differences could be assumed to be meaningful. This necessitates the discriminability of these differences. Consequently, the attractive serial dependence should “make room” for this ability to discriminate by having a more narrow feature tuning width. Alternatively, the repulsive serial dependence component can be strengthened. Both accounts lead to the narrowing of attractive serial dependence feature tuning. The change in serial dependence feature tuning did not replicate in the second experiment of this study, however. Instead, we found a small difference in the maximum magnitude of the attractive serial dependence such that attractive serial dependence was stronger when stimuli were drawn from the full circular distribution than when they were drawn from a narrow uniform sector. This could be due to the data being more noisy, leading to more random variance in the curve fits. The Bayes factor indicated only very moderate evidence for a difference between the two conditions.

## Conclusions

We find that short-, medium-, and long-term effects are separable in orientation judgments. We find no evidence for CTB independent of cardinal bias despite attractive serial dependence. This means that serial dependence does not build up to CTB in orientation judgments, which is in opposition to models of serial dependence and CTB that suggest that they arise from a shared mechanism. Instead, orientation judgments show a medium-term bias away from the mean of the distribution, likely due to adaptation-related mechanisms. Why orientation judgments show a repulsive medium-term bias when other stimulus types cause attractive CTB remains an open question for future research. This study highlights the need for studies and models to consider both attractive and repulsive sequential biases at different timescales.

## Supplementary Material

Supplement 1

Supplement 1
