# Supplementary material for: Serial dependence does not build up to central tendency bias in orientation judgments
Source: J Vis. 2026 Oct 2;26(10):4. doi: 10.1167/jov.26.10.4 (PMC13648532; doi:10.1167/jov.26.10.4)
Supplement: Supplementary file 1 [file jovi-26-10-4_s001.pdf]

Supplement A: Derivative of von Mises

Von Mises distribution is a circular probability distribution that is analogous to the normal distribution. The probability density function of von Mises is

$$f(\theta|\mu,\kappa) = \frac{e^{\kappa\cos(\theta-\mu)}}{2\pi I_0(\kappa)}$$

where θ is an angle in radians (e.g. orientation, hue), μ is the center of the distribution and κ is a measure of concentration. The parameter κ is inversely analogous to variance in a normal distribution. The function $I_0(\kappa)$ is the modified Bessel function of the first kind of order 0. Together with 2π the Bessel function scales the function f such that it satisfies the requirements for a probability density function.

Since we do not need the function to be a probability density function, for our purposes of finding a circular equivalent for the derivate of Gaussian curve, we can use the unscaled version of von Mises. In addition, we can assume that $\mu = 0$.

$$f(\theta|\kappa) = e^{\kappa\cos(\theta)}$$

Differentiating with respect to θ gives

$$f'(\theta|\kappa) = -\kappa\sin(\theta)e^{\kappa\cos(\theta)}$$

We want to give our function the same property as the derivative of Gaussian function used in serial dependence literature has: we want it to have a parameter α that matches the amplitude of the curve. To achieve this, we also need a scaling function $h(\kappa)$. Now, this is our derivative of von Mises function

$$g(\theta|\alpha,\kappa) = h(\kappa)\alpha\kappa\sin(\theta)e^{\kappa\cos(\theta)}$$

We still need to define the scaling function $h(\kappa)$. Differentiate the function g with respect to θ to find maxima and minima:

$$g'(\theta|\alpha,\kappa) = -h(\kappa)\alpha\kappa e^{\kappa\cos(\theta)}\left(\kappa^2\sin^2(\theta) - \cos(\theta)\right)$$

Solve $g'(\theta|\alpha,\kappa) = 0$ assuming that $h(\kappa) \neq 0$ and $\kappa > 0$:

$$-h(\kappa)\alpha\kappa e^{\kappa\cos(\theta)}\left(\kappa\sin^2(\theta) - \cos(\theta)\right) = 0$$

Case 1: $-h(\kappa)\alpha\kappa e^{\kappa\cos(\theta)} = 0$.

We set $h(\kappa) \neq 0$ and $\kappa > 0$ and $e^{\kappa\cos(\theta)}$ can never be 0. Thus this can only be true if $\alpha = 0$. This is a case where the subject has no serial dependence, so it is not interesting.

Case 2: $\kappa\sin^2(\theta) - \cos(\theta) = 0$

$$\begin{aligned}
\kappa\sin^2(\theta) - \cos(\theta) &= 0 \\
\kappa(-\cos^2(\theta) + 1) - \cos(\theta) &= 0 \\
-\kappa\cos^2(\theta) - \cos(\theta) + \kappa &= 0 \\
\cos(\theta) &= \frac{-1 \pm \sqrt{1+4\kappa^2}}{2\kappa} \\
\theta &= \arccos\left(\frac{-1 \pm \sqrt{1+4\kappa^2}}{2\kappa}\right) + n\pi
\end{aligned}$$

We only care about $n = 0$. Thus the maximum is at $\theta = \arccos\left(\frac{-1+\sqrt{1+4\kappa^2}}{2\kappa}\right)$.

Now let $\theta_0 = \arccos\left(\frac{-1+\sqrt{1+4\kappa^2}}{2\kappa}\right)$. We get that the maximum value of derivative of von Mises at θ_0 is

$$\begin{aligned}
g(\theta_0|\alpha,\kappa) &= h(\kappa)\alpha\kappa\sin(\theta_0)e^{\kappa\cos(\theta_0)} \\
&= h(\kappa)\alpha\kappa\sin\left(\arccos\left(\frac{-1+\sqrt{1+4\kappa^2}}{2\kappa}\right)\right)e^{\kappa\cos\left(\arccos\left(\frac{-1+\sqrt{1+4\kappa^2}}{2\kappa}\right)\right)} \\
&= h(\kappa)\alpha\kappa\frac{\sqrt{2\sqrt{1+4\kappa^2}-2}}{2\kappa}e^{\kappa\frac{-1+\sqrt{1+4\kappa^2}}{2\kappa}} \\
&= h(\kappa)\alpha\frac{\sqrt{2\sqrt{1+4\kappa^2}-2}}{2}e^{\frac{-1+\sqrt{1+4\kappa^2}}{2}}
\end{aligned}$$

Now, this must be equal to α. Let's solve for $h(\kappa)$:

$$\begin{aligned}
h(\kappa)\alpha\frac{\sqrt{2\sqrt{1+4\kappa^2}-2}}{2}e^{\frac{-1+\sqrt{1+4\kappa^2}}{2}} &= \alpha \\
h(\kappa)\frac{\sqrt{2\sqrt{1+4\kappa^2}-2}}{2}e^{\frac{-1+\sqrt{1+4\kappa^2}}{2}} &= 1 \\
h(\kappa) &= \frac{2}{\sqrt{2\sqrt{1+4\kappa^2}-2}e^{\frac{-1+\sqrt{1+4\kappa^2}}{2}}} \\
h(\kappa) &= \frac{2e^{\frac{1-\sqrt{1+4\kappa^2}}{2}}}{\sqrt{2\sqrt{1+4\kappa^2}-2}} \\
h(\kappa) &= \frac{\sqrt{2}e^{\frac{1-\sqrt{1+4\kappa^2}}{2}}}{\sqrt{\sqrt{1+4\kappa^2}-1}} \\
h(\kappa) &= \sqrt{\frac{2}{\sqrt{1+4\kappa^2}-1}}e^{\frac{1-\sqrt{1+4\kappa^2}}{2}}
\end{aligned}$$

In conclusion, the derivative of von Mises function is

$$DovM(\theta|\alpha,\kappa) = h(\kappa)\alpha\kappa\sin(\theta)e^{\kappa\cos(\theta)}$$

where θ is the orientation difference, α is the maximum amplitude of the curve, κ is the measure on concentration nad $h(\kappa)$ is a scaling function where

$$h(\kappa) = \sqrt{\frac{2}{\sqrt{1+4\kappa^2}-1}}e^{\frac{1-\sqrt{1+4\kappa^2}}{2}}.$$
