# Supplementary material for: Serial dependence does not build up to central tendency bias in orientation judgments
Source: J Vis. 2026 Oct 2;26(10):4. doi: 10.1167/jov.26.10.4 (PMC13648532; doi:10.1167/jov.26.10.4)
Supplement: Supplementary file 2 [file jovi-26-10-4_s002.pdf]

Supplement B

Simulation: Orthogonalizing removes the effect of serial dependence from CTB

```
rng(0)
```

We simulate one observer with an attractive serial dependence when the preceding stimulus is similar to the current one (attractive component) and a strong repulsive serial bias for stimuli that differ more (repulsive component).

Stimuli are random orientations between 10-80 degrees.

```
N = 5000;
stim = randi([10, 80], [1, N]);
previous_stim = [45, stim(1:end-1)];
delta = circular_difference(previous_stim, stim, 180);
```

The simulated observer gives a response as a weighted sum of the current stimulus and previous stimuli such that the weights depend on the similarity (δ) of current and previous stimulus. Additionally, the response has random noise (here with a normal distribution). The response error is then:

```
resp_error = dovm(delta, 3, 8, 180) + dovm(delta, -4.5, 0.1, 180) + randn([1,
N]);
```

Now, we plot both central tendency bias (CTB) and serial dependence without controlling for anything.

```
figure()
tiledlayout(1,2)

nexttile
hold on
scatter(stim, resp_error,'ok','filled','MarkerFaceAlpha',0.05)
CTBfit = polyfit(stim, resp_error, 1);
plot(linspace(10,80), polyval(CTBfit,linspace(10,80)), 'LineWidth', 3)
xlabel('Stimulus')
ylabel('Response error')
title('"CTB"')

nexttile
scatter(delta, resp_error,'ok','filled','MarkerFaceAlpha',0.05)
hold on
x = linspace(-70,70);
plot(x,dovm(x, 3, 8, 180) + dovm(x, -4.5, 0.1, 180), 'LineWidth', 3)
xlim([-90,90])
xlabel('Previous - current stimulus')
ylabel('Response error')
title('Serial dependence')
```

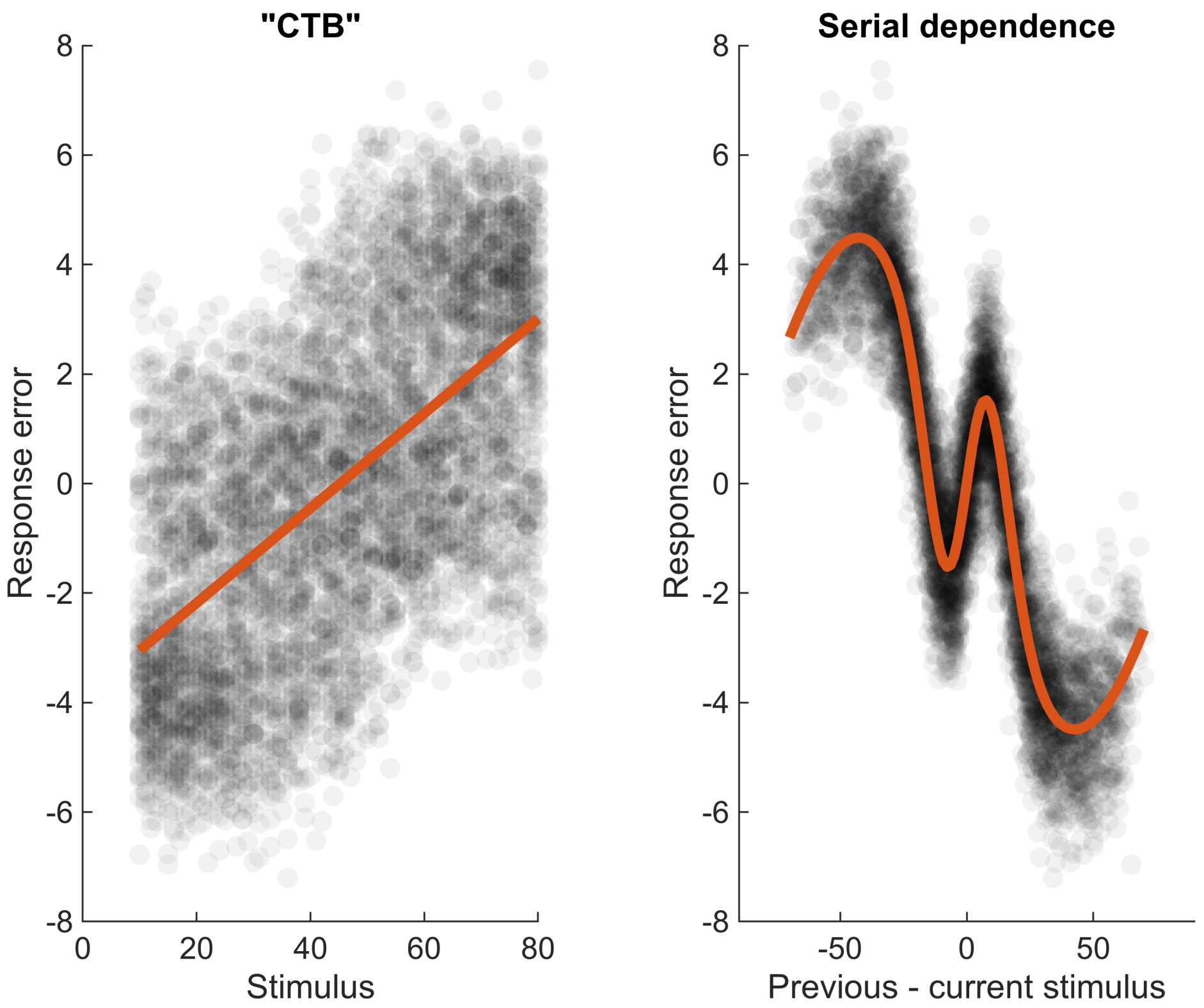


```
display(join(["CTB slope: " CTBfit(1)]))
```

```
"CTB slope:  0.086528"
```

This could be interpreted as a general bias away from the center of the stimulus distribution. However, this is incorrect as the observer has no bias caused by the stimulus distribution itself.

Next, we orthogonalize the vector "`stim`" with respect to the vector "`delta`" using the Gram–Schmidt process.

```
orth_stim = stim - (dot(stim, delta) / dot(delta, delta)) * delta;
```

Make the same figures as above.

```
figure()
tiledlayout(1,2)

nexttile
hold on
scatter(orth_stim, resp_error,'ok','filled','MarkerFaceAlpha',0.05)
CTBfit = polyfit(orth_stim, resp_error, 1);
plot(linspace(10,80), polyval(CTBfit,linspace(10,80)), 'LineWidth', 3)
xlabel('Orthogonalized stimulus')
ylabel('Response error')
title('"CTB"')

nexttile
scatter(delta, resp_error,'ok','filled','MarkerFaceAlpha',0.05)
hold on
```

```
x = linspace(-70,70);
plot(x,dovm(x, 3, 8, 180) + dovm(x, -4.5, 0.1, 180), 'LineWidth', 3)
xlim([-90,90])
xlabel('Previous - current stimulus')
ylabel('Response error')
title('Serial dependence')
```

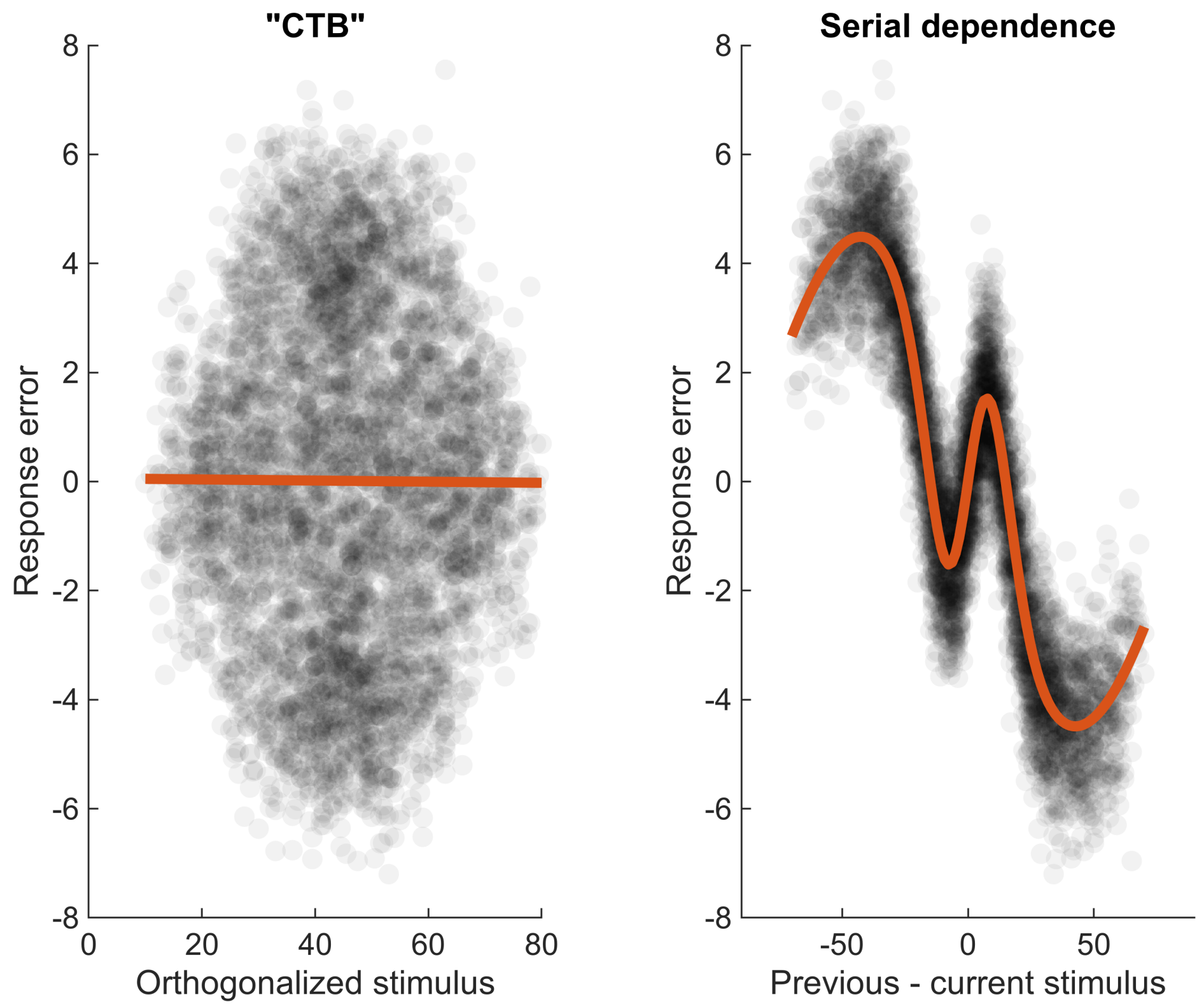


```
display(join(["CTB slope: " CTBfit(1)]))
```

```
"CTB slope:  -0.0010365"
```

The slope is now correctly 0 which reflects the fact that the biases are entirely caused by the preceding stimulus and not the whole stimulus distribution.

Help functions

```
function diff = circular_difference(a, b, full_circle)
  diff = mod((a-b) + full_circle/2, full_circle) - full_circle/2;
end

function y = dovm(x, alpha, kappa, degmax)
    theta = (2 * pi * x) / degmax;
    c = (sqrt(2) * exp((1 - sqrt(1 + 4 * kappa.^2)) / 2)) ./ sqrt(sqrt(1 + 4 *
kappa.^2) - 1);
    y = c .* alpha .* kappa .* sin(theta) .* exp(kappa .* cos(theta));
end
```
